# Evaluation of auto-segmentation for EBRT planning structures using deep learning-based workflow on cervical cancer

**DOI:** 10.1038/s41598-022-18084-0

**Published:** 2022-08-11

**Authors:** Jiahao Wang, Yuanyuan Chen, Hongling Xie, Lumeng Luo, Qiu Tang

**Affiliations:** Department of Radiation Oncology, Women’s Hospital, School of Medicine, Zhejiang University, Hangzhou, 310006 Zhejiang China

**Keywords:** Radiotherapy, Cancer imaging

## Abstract

Deep learning (DL) based approach aims to construct a full workflow solution for cervical cancer with external beam radiation therapy (EBRT) and brachytherapy (BT). The purpose of this study was to evaluate the accuracy of EBRT planning structures derived from DL based auto-segmentation compared with standard manual delineation. Auto-segmentation model based on convolutional neural networks (CNN) was developed to delineate clinical target volumes (CTVs) and organs at risk (OARs) in cervical cancer radiotherapy. A total of 300 retrospective patients from multiple cancer centers were used to train and validate the model, and 75 independent cases were selected as testing data. The accuracy of auto-segmented contours were evaluated using geometric and dosimetric metrics including dice similarity coefficient (DSC), 95% hausdorff distance (95%HD), jaccard coefficient (JC) and dose-volume index (DVI). The correlation between geometric metrics and dosimetric difference was performed by Spearman’s correlation analysis. The right and left kidney, bladder, right and left femoral head showed superior geometric accuracy (DSC: 0.88–0.93; 95%HD: 1.03 mm–2.96 mm; JC: 0.78–0.88), and the Bland–Altman test obtained dose agreement for these contours (*P* > 0.05) between manual and DL based methods. Wilcoxon’s signed-rank test indicated significant dosimetric differences in CTV, spinal cord and pelvic bone (*P* < 0.001). A strong correlation between the mean dose of pelvic bone and its 95%HD (R = 0.843, *P* < 0.001) was found in Spearman’s correlation analysis, and the remaining structures showed weak link between dosimetric difference and all of geometric metrics. Our auto-segmentation achieved a satisfied agreement for most EBRT planning structures, although the clinical acceptance of CTV was a concern. DL based auto-segmentation was an essential component in cervical cancer workflow which would generate the accurate contouring.

## Introduction

External beam radiation therapy (EBRT) and brachytherapy (BT) are both the critical treatment modalities for cervical cancer with early and locally advanced stages. The delineation of clinical target volumes (CTVs) and organs at risk (OARs) is the first step and important task that may affect the clinical outcomes in cervical cancer radiotherapy^1–3^. Indeed, manual contouring of these planning structures is such a labor-intensive part of the workflow and maybe inaccurate^4–6^. The workload pressures and most errors could be avoided if a rapid and accurate auto-segmented methods were available. With the development of machine learning (ML), particularly the advent of deep learning (DL) represented by convolutional neural networks (CNNs), auto-segmented tasks are thought to provide excellent assistance and superior results^7–10^.

The U-Net model used for auto-segmentation of OARs in cervical cancer obtained highly consistency with those of expert contouring which was assessed by radiation oncologists^11^. The DpnUNet model applied to CTV segmentation in cervical cancer achieved an acceptable clinical results with the mean dice similarity coefficient (DSC) of 0.86^12^. As a novel technique, however, there are still some limitations with uncommon clinical practice^13^. In fact, DL based methods are usually to generate the expected outcomes because the tested datasets are typically related to the training and validating datasets. Therefore, the quality and reliability of DL models should be further verified using an independent cohort in the process of cervical cancer radiotherapy.

The geometric metrics and subjective assessment are always chosen as the standard analysis indicators of contour comparison^14–16^. A few studies have reported the relationship between auto-segmentation and dosimetry in head and neck which proved more accurate auto-segmentation carried out smaller dosimetric differences^17^. However,whether or not the differences of DL based auto-segmentation would affect the clinical relevance of cervical cancer is rarely mentioned.

The purpose of this study used geometric and dosimetric metrics to evaluate the accuracy of DL based auto-segmentation and focus on the question of whether DL based approach could generate precise dosimetric endpoints compared to standard manual contours in a real-world independent cohort of cervical cancer patients.

## Methods and materials

### Experiments

**Figure 1:**
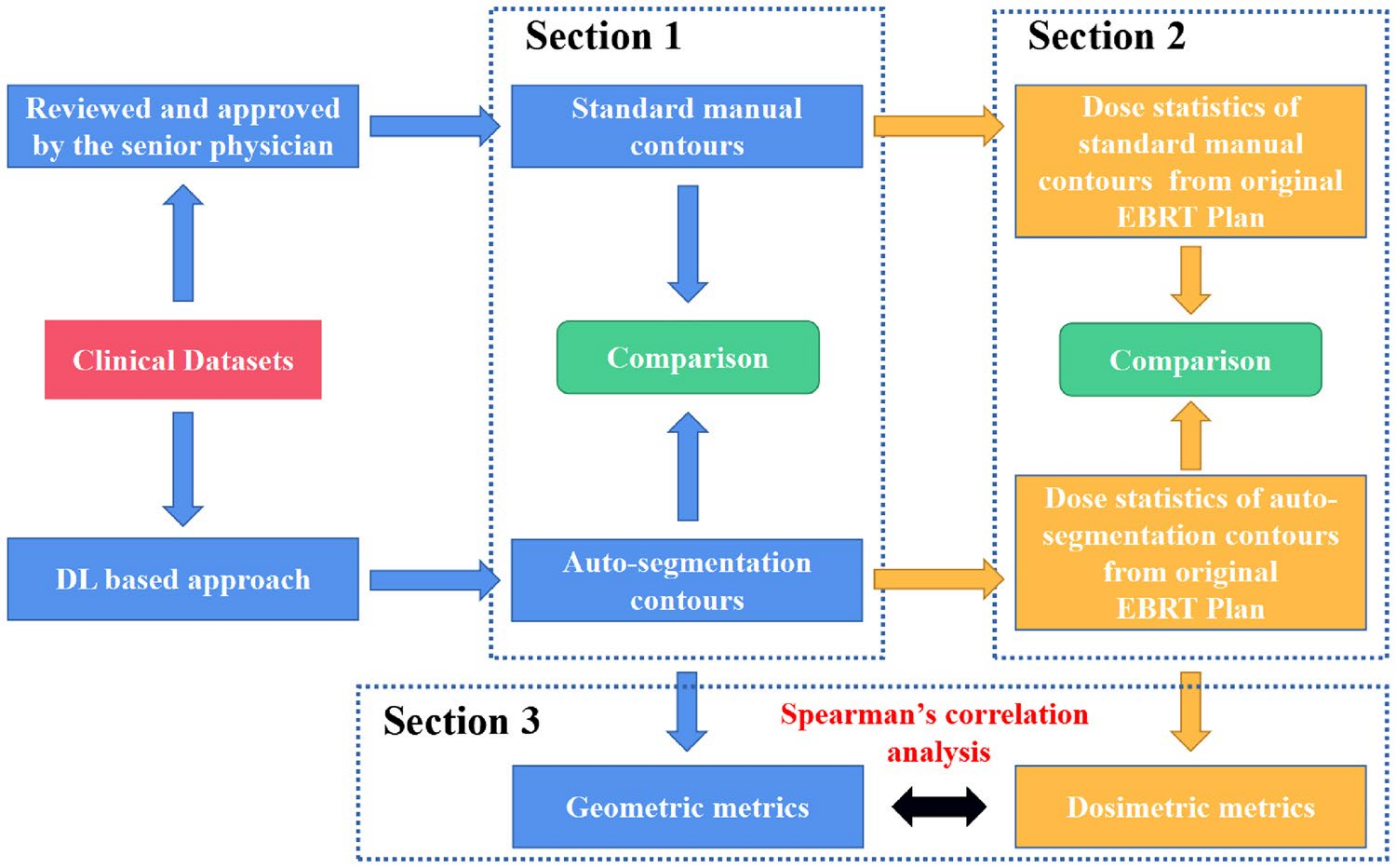
The flowchart of manual and DL based auto-segmentation evaluation experiment. Original EBRT plans were designed and optimized based on the standard manual contours and the auto-segmentation structures were transmitted to original EBRT plans for dosimetric evaluation.

The work flowchart of this study is illustrated in Fig. 1. Briefly, the evaluation was divided into 3 sections. Section 1, the accuracy of DL based auto-segmentation was assessed using geometric metrics. Section 2, the dosimetric comparison was performed between standard manual contours and auto-segmented contours form original EBRT plans. Section 3, the correlation analysis was explored followed by geometric and dosimetric metrics.

### Clinical datasets

The independent cohort of this study was consisted of 75 cervical cancer patients who received EBRT at our department between August 2021 and December 2021. All patients were diagnosed with FIGO stage IA2-IVB and histology G1-G3, treated with prescription dose of 45 Gy-50.4 Gy (1.8 Gy/fraction). The average age ± standard deviation of these patients was 55.60 ± 13.35 years old. For each patient, the contrast agent was required to intravenously inject before computed tomography (CT) scanning, meanwhile, the CT images were covered from the lower lumbar spine to the whole pelvic cavity and reconstructed with 512 × 512 matrix size and 5 mm slice thickness using a Philips Brilliance Big Bore CT scanner system (Philips Healthcare,Best, the Netherlands).

CTVs delineation of 75 patients were defined manually by junior radiation oncologists including entire cervix, uterus, bilateral parametria, upper half of vagina, and lymph nodes following the guideline of Radiation Therapy Oncology Group (RTOG) protocol^18^. Relevant OARs included for EBRT plans were spinal Cord, left kidney (Kidney L), right kidney (Kidney R),bladder, left femoral Head (Femoral Head L), right femoral Head (Femoral Head R), pelvic bone, rectum, and small intestine. The EBRT planning structures were performed on the Pinnacle Treatment Planning System (Pinnacle, V9.16.2, Philips Corp, Fitchburg, WI, USA). All of the manual contours were reviewed and approved by senior radiation oncologists specialized in cervical cancer to generate the standard delineation.

### Deep learning based auto-segmentation

**Figure 2:**
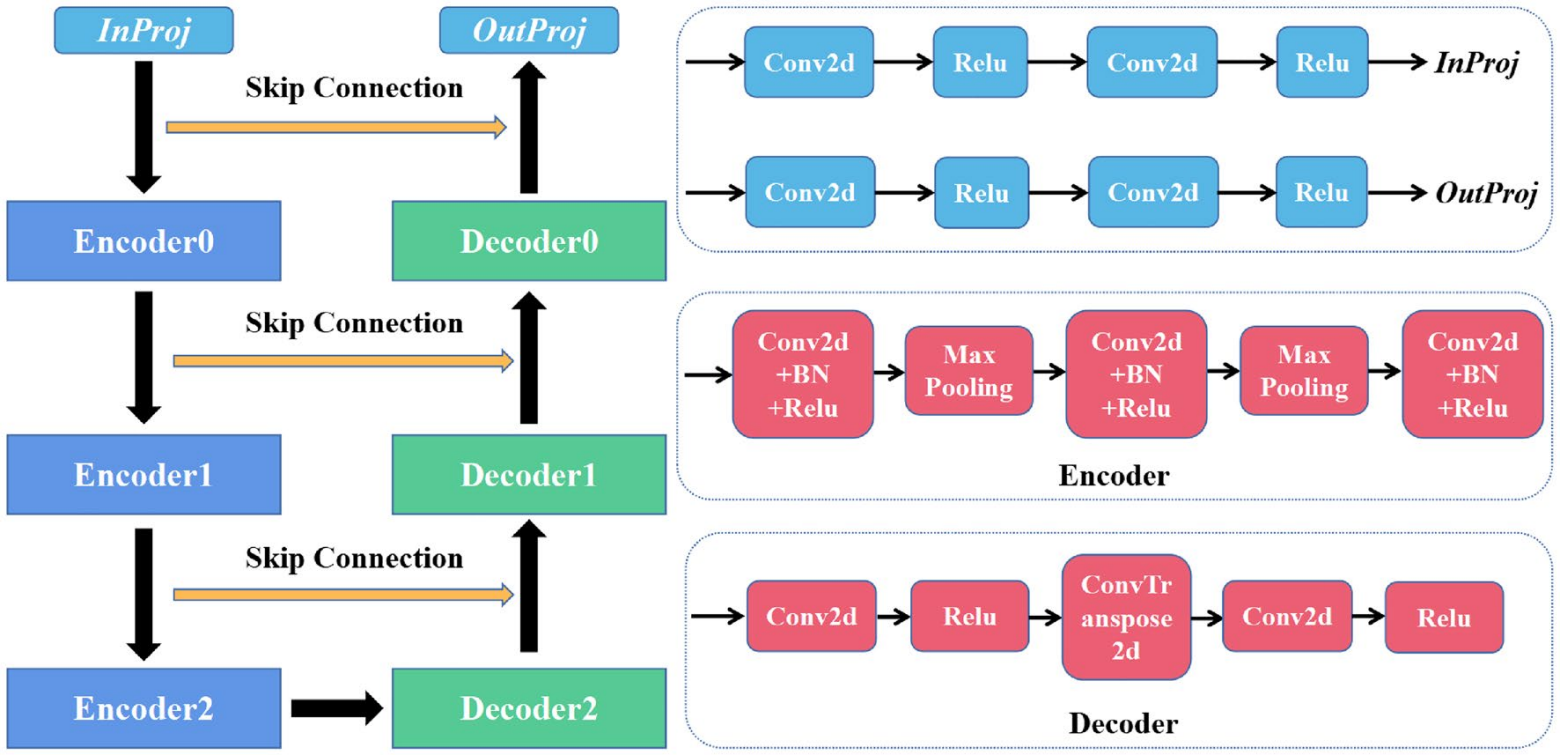
Architecture of DL based automatic segmentation network.

We introduced a deep learning model based on CNN^19^ to segment the CTVs and OARs for cervical cancer patients. As shown in Fig. 2, the network consists of three encoders and three decoders. The InProj was used to extract the features of medical image, and the OutProj performed the pixel-wise classification. Down-sampling and up-sampling were performed by each encoder and each decoder. All the weight filters of the 2D convolution (Conv2d) had a window size of 3 × 3 and a stride of 1. Batch Normalization (BN) was a process by which biased output distribution and used for the feature normalization. For this network, rectified linear unit (ReLu) followed by every Conv2d was used as the feature activation function. Max Pooling could reduce the number of parameters and computation in the network. ConvTranspose2d was opposite of that used for Conv2d, whereby pixel size is increased using a 3 × 3 pixels filter. The skip connection was used to concatenate the encoder and decoder of the same level to facilitate the fusion of multi-layer features. We used some general methods for data enhancement (cut and flip) to obtain a superior model. This model is an end-to-end segmentation architecture that can predict pixel class labels in CT images.

A total of 300 retrospective clinical CT scans diagnosed with cervical cancer who received radiotherapy were enrolled for training and validating this model, and the datasets were come form multiple cancer centers in order to verify the robustness of CNN model. The cross-entropy loss was selected as the loss function, and all of the training computations were performed using Intel-Core i7 processor with a graphics card.

### Geometric metrics

The geometric accuracy of contours was compared using the Dice Similarity Coefficient (DSC), 95% Hausdorff Distance (HD) and Jaccard Coefficient (JC). DSC and JC describe the relative overlap between segmentation A and B. HD is used to quantify the 3D distance between two segmentation surfaces. The 95%HD is the distance that indicates the largest surface-to-surface separation among the closest 95% of surface points.The definitions are as follows:

$$\begin{aligned} & DSC = 2\left| {A \cap B} \right|/(\left| A \right| + \left| B \right|) \\ & HD = \max (h(A,B),h(B,A)),\;h(A,B) = \mathop {\max }\limits_{b \in B} (\mathop {\min }\limits_{a \in A} \left\| {a - b} \right\|) \\ & JC = \left| {A \cap B} \right|/\left| {A \cup B} \right| \\ \end{aligned}$$

For the complete overlap, the value of HD is 0, and the values of DSC and JC are 1. For the incomplete overlap, the value of HD is large, and the values of DSC and JC are close to 0. In order to verify the recognition performance of DL based model in boundary of segmentation,no cropping of the superior or inferior borders for contours was performed for this study particularly in spinal cord, femoral head and pelvic bone.

### Dosimetric metrics

The EBRT plans were calculated and optimized with these standard manual contours by using Pinnacle Treatment Planning System. Table 1 is presented the constraints and dosimetric metrics. For CTV, we mainly focused on D_mean_ and V_100%_. For serial organs and parallel organs, we mainly focused on D_max_ and D_mean_, respectively. D_mean_ and D_max_ are defined as the average dose and maximum dose of structures receiving. V_100_ is defined as the volume of CTV receiving 100% prescription dose.

**Table 1:** The constraints and dosimetric metrics for EBRT planning structures.

Structures	Constraints	Dosimetric metrics
CTV	D_99%_ > Prescription, D_max_ < 110%Prescription	D_mean_, V_100_
Spinal Cord	D_max_ < 4000 cGy	D_max_
Kidney L	D_mean_ < 1200 cGy	D_mean_
Kidney R	D_mean_ < 1200 cGy	D_mean_
Bladder	D_50%_ < 100%Prescription D_0.03 cc_ < 110%Prescription	D_mean_
Femoral Head L	D_15%_ < 3000 cGy,Mean dose < 2000 cGy	D_mean_
Femoral Head R	D_15%_ < 3000 cGy,Mean dose < 2000 cGy	D_mean_
Pelvic Bone	D_mean_ < 3000 cGy	D_mean_
Rectum	D_50%_ < 100%Prescription D_0.03 cc_ < 110%Prescription	D_mean_
Small intestine	D_30%_ < 100%Prescription D_0.03 cc_ < 110%Prescription	Not evaluated

*CTV:* clinical target volume; *Kidney L/R:* left/right kidney; *Femoral Head L/R:* left/right femoral head.

### Statistical analysis

IBM SPSS Statistics software (version 19.0, IBM Inc., Armonk, NY, USA) and Python software (version 3.6.5,Anaconda Inc.) were used for statistical analysis,where mean ± standard deviation (SD) was used for presenting and summarizing the results. For the test of agreement between manual and DL based methods, the Bland–Altman test was used to calculate the consistent limits for each EBRT planning structures. *P* > 0.05 means agreement of two segmented methods. For the difference, the Wilcoxon’s paired nonparametric signed-rank test was performed to compare the variables. *P* < 0.05 indicates that the difference is statistically significant. The correlations between geometric metrics and dosimetric difference were evaluated with Spearman’s correlation analysis.

## Results

**Figure 3:**
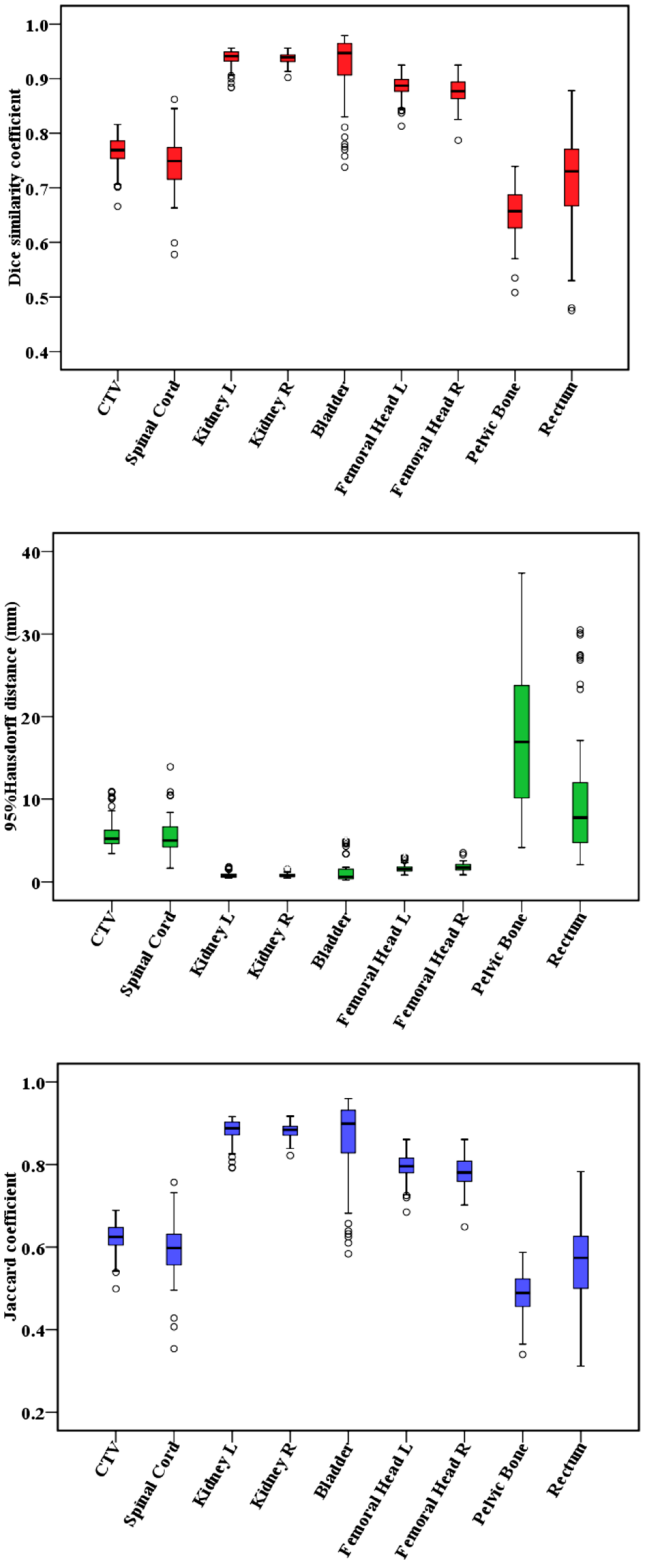
DSC, 95%HD and JC box plot from comparing DL based auto-segmented contours to standard contours for CTV and OARs.

The geometric accuracy of the DL based auto-segmentation for EBRT planning structures is presented in Fig. 3. Automatic delineation produced the results for CTV with average DSC value of 0.77 ± 0.03, 95%HD of 5.81 ± 1.83 mm and JC of 0.62 ± 0.04. The right kidney, left kidney, bladder, right femoral head and left femoral head were generated the similar geometric performance between two methods with average DSC value of 0.88–0.93, 95%HD of 1.03–2.96 mm and JC of 0.78–0.88. The quality of the automatically generated pelvic bone was barely satisfactory with average DSC value of 0.65 ± 0.05,95%HD of 18.14 ± 9.77 mm and JC of 0.49 ± 0.05.

**Figure 4:**
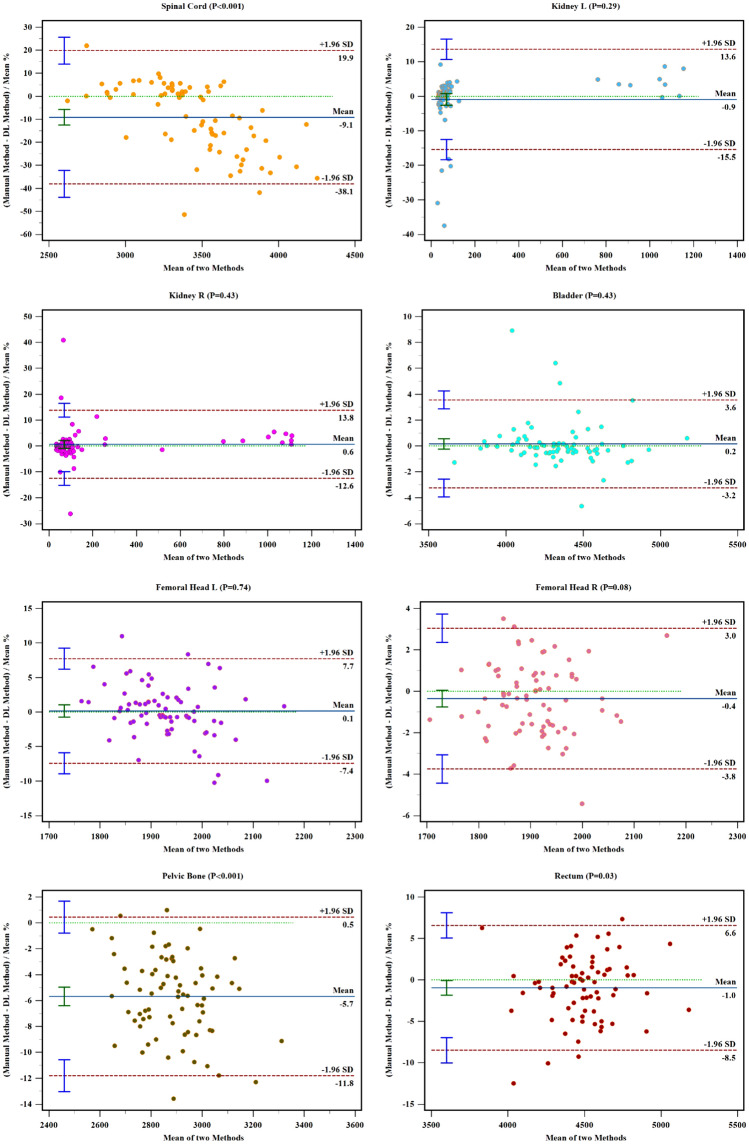
Bland–Altman plot for OARs. The brown horizontal dotted lines represents the upper and lower bounds of 95% limit agreement; the blue horizontal solid lines represent the average of the differences; the green horizontal dotted lines represent the location with difference equal to 0.

The Bland–Altman test was not calculated for CTV because of abnormal distribution. The Fig. 4 showed 95% consistent limits for all of the OARs between two methods. The test of agreement for DL based auto-segmentation method can be evaluated according to the number of the points outside the 95% consistent limits (brown horizontal dotted lines) and the maximum difference within the consistent limits (distance between blue and green horizontal lines). From the Bland–Altman plot, right and left kidney, bladder, right and left femoral head showed no significant inconsistency (*P* > 0.05) between two segmented methods.

**Figure 5:**
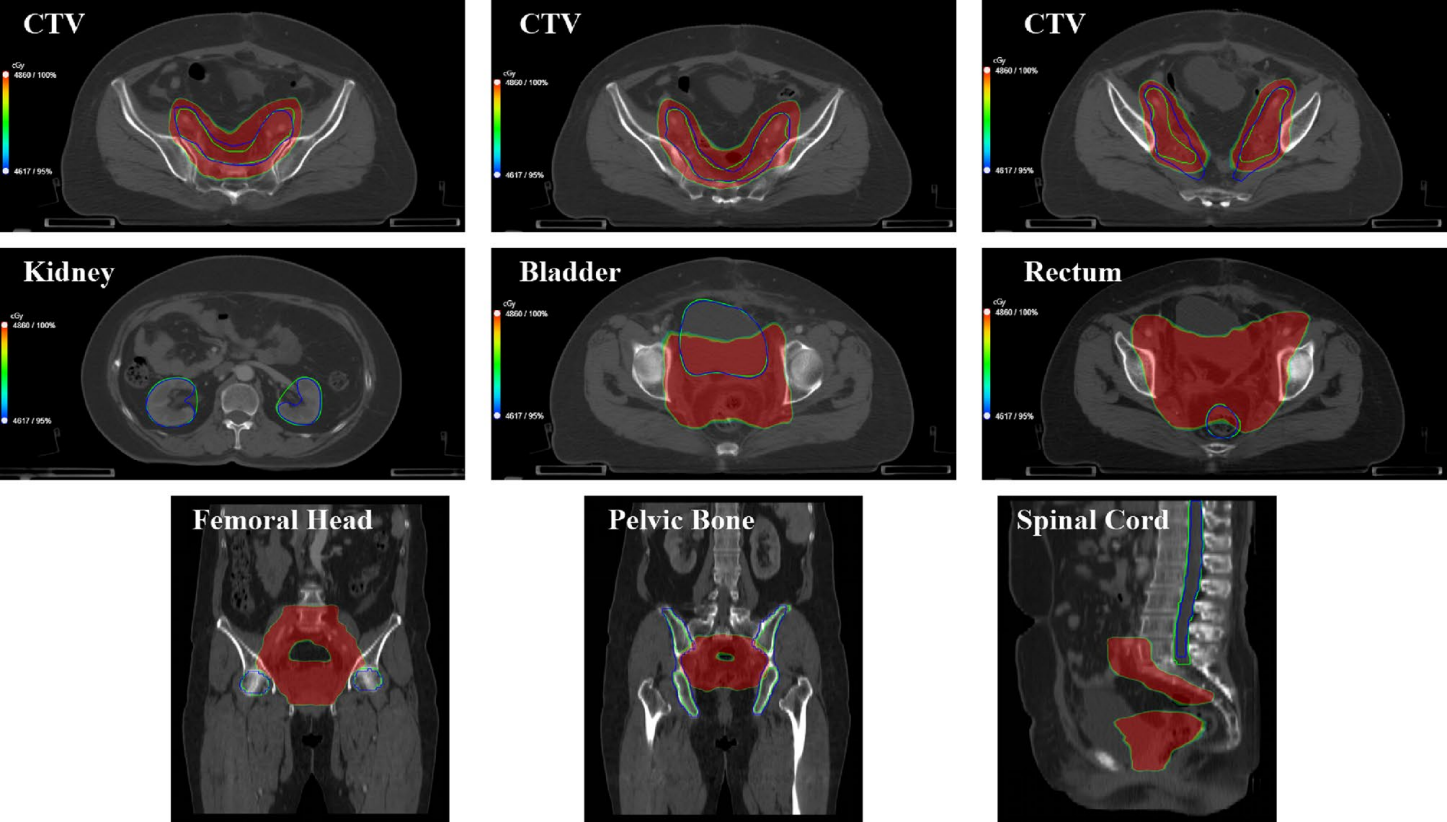
Results of delineations and dose distributions for CTV and OARs in CT slices. The green lines represent manual contours approved by the senior physician; the blue lines represent DL based contours; colourwash represent dose distributions with the range of 95% prescription to 100% prescription.

Examples of delineations and dose distributions from manual and DL based auto-segmented methods are illustrated in Fig. 5. The comparisons of dosimetric parameters between two methods using Wilcoxon’s paired nonparametric signed-rank test are presented in Table 2. No significant dosimetric differences were found except for CTV, spinal cord and pelvic bone (*P* < 0.001). For all of the OARs, both the manual and automatic delineation were able to meet the clinical dose constraints. However, the dose-volume index (DVI) of CTV was hard to meet the clinical requirements with V_100_ (%) of 94.27 ± 1.86 (D_99%_ > Prescription).

**Table 2:** Dosimetric metrics of manual and DL based auto-segmented delineations in the original clinical treatment plans.

Structure	Dosimetric parameters	Manual delineation	Automatic delineation	Z	*P*
		Mean ± standard deviation			
CTV	D_mean_ (cGy)	5058.71 ± 191.85	4972.37 ± 194.18	− 7.53	< 0.001
	V_100_ (%)	99.98 ± 0.02	94.27 ± 1.86	− 7.53	< 0.001
Spinal Cord	D_max_ (cGy)	3270.17 ± 259.72	3616.79 ± 565.83	− 4.00	< 0.001
Kidney L	D_mean_ (cGy)	175.41 ± 320.37	171.29 ± 306.65	− 1.67	0.096
Kidney R	D_mean_ (cGy)	205.68 ± 318.12	201.80 ± 308.71	− 0.99	0.323
Bladder	D_mean_ (cGy)	4345.07 ± 263.11	4338.67 ± 270.32	0.95	0.342
Femoral Head L	D_mean_ (cGy)	1930.73 ± 74.68	1928.89 ± 97.26	− 0.48	0.631
Femoral Head R	D_mean_ (cGy)	1897.44 ± 75.49	1901.13 ± 84.13	− 1.24	0.085
Pelvic Bone	D_mean_ (cGy)	2802.99 ± 129.23	2968.00 ± 160.85	− 7.48	< 0.001
Rectum	D_mean_ (cGy)	4490.24 ± 252.17	4523.57 ± 248.61	− 1.61	0.108

**Figure 6:**
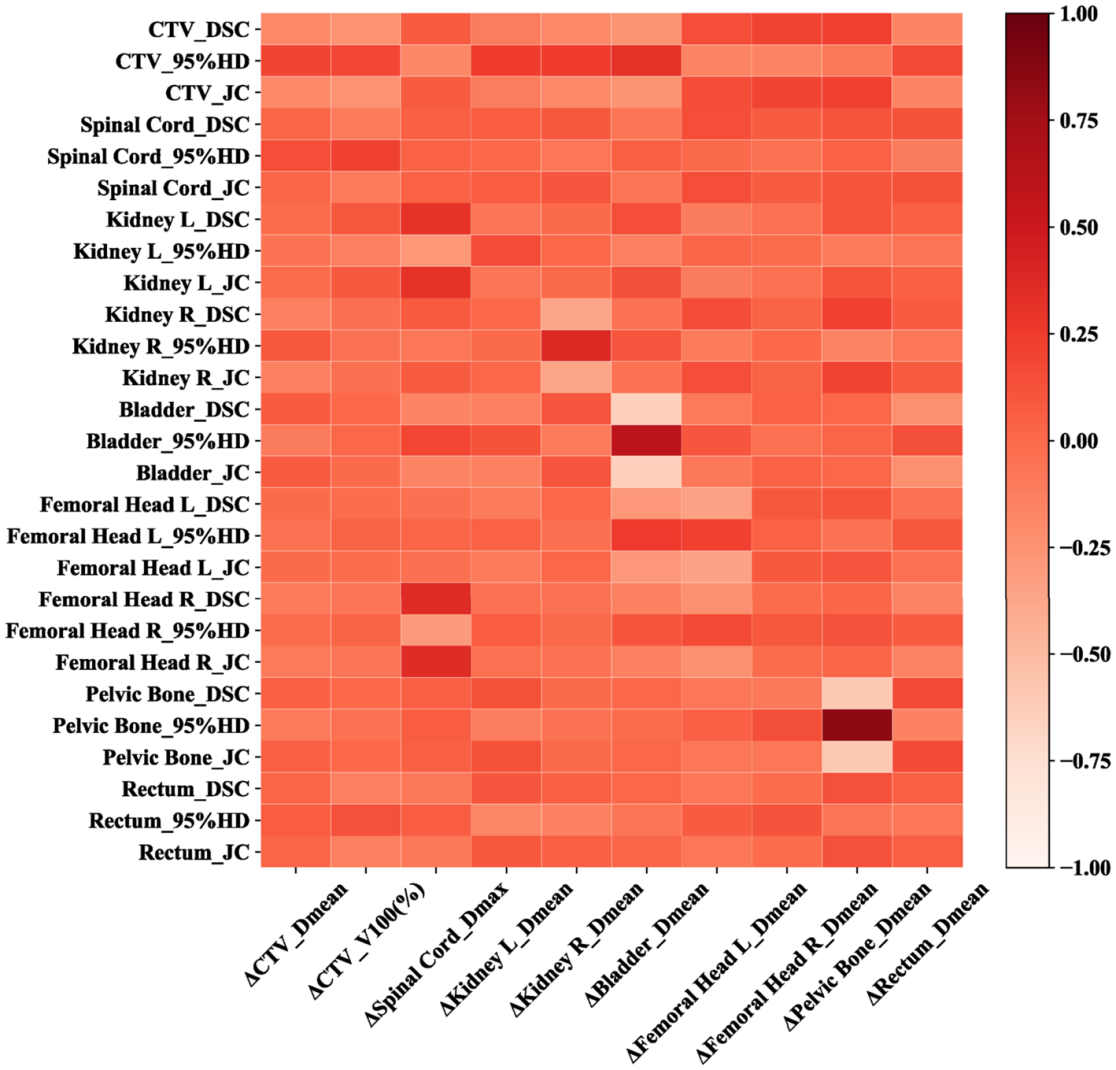
The heatmap of Spearman’s correlation analysis between all the geometric metrics and dosimetric differences for EBRT planning structures.

Table 3 shows the results of Spearman’s correlation analysis between three geometric metrics and dosimetric differences (Δdose). No structures showed strong correlation except for the ΔD_mean_ of pelvic bone and its 95%HD (R = 0.843,*P* < 0.001), and the correlation heatmap was used to further prove the weak link between all of the dosimetric difference and its geometric metrics in the remaining EBRT planning structures (Fig. 6).

**Table 3:** The correlation between geometric metrics and dosimetric differences.

Structure	ΔDose	Geometric metrics	Correlation analysis
CTV	ΔD_mean_	DSC	R = − 0.198, *P* = 0.089
		95%HD	R = 0.089, *P* = 0.087
		JC	R = − 0.195, *P* = 0.093
CTV	ΔV_100_ (%)	DSC	R = − 0.245, *P* = 0.034
		95%HD	R = 0.180, *P* = 0.123
		JC	R = − 0.245, *P* = 0.034
Spinal Cord	ΔD_max_	DSC	R = 0.047, *P* = 0.688
		95%HD	R = 0.046, *P* = 0.694
		JC	R = 0.043, *P* = 0.711
Kidney L	ΔD_mean_	DSC	R = − 0.076, *P* = 0.518
		95%HD	R = 0.162, *P* = 0.166
		JC	R = − 0.074, *P* = 0.528
Kidney R	ΔD_mean_	DSC	R = − 0.361, *P* = 0.001
		95%HD	R = 0.379, *P* = 0.001
		JC	R = − 0.354, *P* = 0.002
Bladder	ΔD_mean_	DSC	R = − 0.644, *P* < 0.001
		95%HD	R = 0.601, *P* < 0.001
		JC	R = − 0.646, *P* < 0.001
Femoral Head L	ΔD_mean_	DSC	R = − 0.341, *P* = 0.003
		95%HD	R = 0.225, *P* = 0.052
		JC	R = − 0.349, *P* = 0.002
Femoral Head R	ΔD_mean_	DSC	R = − 0.014, *P* = 0.902
		95%HD	R = 0.095, *P* = 0.418
		JC	R = − 0.015, *P* = 0.899
Pelvic Bone	ΔD_mean_	DSC	R = − 0.588, *P* < 0.001
		95%HD	R = 0.843, *P* < 0.001
		JC	R = − 0.589, *P* < 0.001
Rectum	ΔD_mean_	DSC	R = 0.054, *P* = 0.648
		95%HD	R = − 0.082, *P* = 0.482
		JC	R = 0.055, *P* = 0.641

*DSC:* dice similarity coefficient; *HD:* hausdorff distance; *JC*: jaccard coefficient; *ΔDose*: dosimetric differences between two segmented methods.

## Discussion

Modern radiotherapy has become a systematized and programmed process resulting in a nearly reliance on human–machine interactions with the development of mechanical technology and computer science. Meanwhile, the growth of Artificial intelligence (AI) has the potential possibilities to change the way of radiation oncology because of its recognition and analysis in complex medical data. Various studies have investigated the advantages of AI based method during each stage of radiotherapy,such as AI platforms might improve the efficiency and quality of automated segmentation^20–22^, predict and optimize the radiation dose of the targets^23,24^, provide the clinical decision of radiation toxicities^25^, and build the robust models to manage the treatment outcomes^26,27^. However, these studies were always fragmented and we should establish the complete radiotherapy workflow using AI technology with validating every step for the real-world cohort.

Delineations of CTV and OARs are an essential step for precise delivery^28^ which would affect the overall survival in the radiotherapy treatment planning process,even in standardizing clinical trials^29^. However, the manual process always suffers from inter- and intra-observer variability in structure delineations. Automatic contouring of structures is highly desired in radiotherapy because of the minimized variability. The purpose of this study is to compare the performance of DL based autosegmentation against standard contours from senior radiation oncologists on independent datasets.

As for geometric metrics, we observed that DL based model generated structures with average DSC of 0.77 for the CTV, 0.74 the spinal cord, 0.93 for the left and right kidney, 0.91 for the bladder, 0.88 for the left and right femoral head, 0.65 for the pelvic bone, and 0.71 for the rectum, respectively. The comparison of DSC and HD for other DL based model is presented in Table 4. Overall, the geometric similarity of kidney, bladder and femoral head were equivalent to or better than other published literature. Nevertheless, the DSC values of CTV, pelvic bone and rectum from our model showed poor results compared with other DL based models. Generally, the accuracy maybe decrease when using the independent testing datasets. Rhee et al.^30^ reported the DSC values of automatic CTV segmentation was 0.86 using internal test CT scans and the clinical acceptance decreased to 80% for external test CT scans. However, the mean 95%HD value of CTV used our model was 5.81 mm, which was comparable to DpnUNet model^12^ and superior than 3D CNN and 3D V-Net models^31,32^. These findings seemed to indicate that the discrepancy between these DL based models might caused by the difference of training datasets, and our DL based model showed a relative strong robustness for most EBRT planning structures enrolled the independent cohort. In this study, the boundaries of the spinal cord in cervical cancer were not clear (the resolution of soft tissue in CT images was deficient and we didn’t modify the superior or inferior borders), the delineations generated by DL based model were always been overestimated or underestimated compared with standard contours. The small intestine was absent to assess because the contours of the small intestine in CT images was different from the location during EBRT process. Indeed, small intestine is an important organ for dosimetric evaluation especially in the EBRT combined with high-dose rate BT for cervical cancer, and the DL based performance of small intestine would be included in our further study with “dose prediction”.

**Table 4:** Summary of DL based auto-segmentation results for CTV and OARs in cervical cancer from other published literature.

Group	DL model	Enrolled patients	Structures	DSC	HD(mm)	
Wang et al.^31^	3D CNN	125 cases from the First Affiliated Hospital of Anhui Medical University in China; 25 internal cases for testing	CTV	0.86	14.84	^31^
			Bladder	0.91	7.82	
			Femoral Head L	0.88	6.17	
			Femoral Head R	0.88	6.18	
			Rectum	0.81	7.04	
			Small intestine	0.86	22.21	
Liu et al.^11^	DpnU-Net	237 cased from Peking Union Medical College Hospital in China; 27 internal cases for testing	CTV	0.86	5.34	^12^
			Spinal Cord	0.82	4.96	
			Bladder	0.91	4.05	
			Femoral Head L	0.90	1.27	
			Femoral Head R	0.90	1.51	
			Bone marrow	0.85	2.16	
			Rectum	0.82	4.29	
Rhee et al.^30^	V-Net + Modified FCN-8 s	2254 female pelvic CT scans from MD Anderson Cancer Center in USA; 140 internal cases and 30 independent cases for testing	CTV	0.85	2.02	^30^
			Spinal cord	0.90	0.65	
			Kidney L	0.94	0.76	
			Kidney R	0.95	0.84	
			Bladder	0.89	1.07	
			Femoral Head L	0.94	0.60	
			Femoral Head R	0.93	0.66	
			Pelvic bone	0.93	1.06	
			Rectum	0.80	1.66	
Ding et al.^32^	3D V-Net	130 cases from Hubei Cancer Hospital in China; 30 internal cases for testing	CTV	0.85	11.2	^32^
			Spinal cord	0.73	2.26	
			Kidney L	0.92	4.54	
			Kidney R	0.92	4.05	
			Bladder	0.94	4.52	
			Femoral Head L	0.82	7.62	
			Femoral Head R	0.81	11.72	
			Pelvic bone	0.92	5.82	
			Rectum	0.85	4.35	
Our method	Modified CNN	300 cases from multiple cancer centers in China; 75 independent cases for testing	CTV	0.77	5.81	
			Spinal cord	0.74	7.42	
			Kidney L	0.93	1.03	
			Kidney R	0.93	1.12	
			Bladder	0.91	2.09	
			Femoral Head L	0.88	2.96	
			Femoral Head R	0.88	2.35	
			Pelvic bone	0.65	18.43	
			Rectum	0.71	10.01	

*CNN:* convolutional neural network; *DL model:* deep learning model.

The quality of auto segmented contours cannot be determined only by geometric values which was reported by Kaderka^33^, and few studies have focused on dosimetric impact on the automatic CTV and OARs delineations for cervical cancer radiotherapy. For CTV dosimetric metrics, the most significant dose difference was V_100_ with 94.27% for DL based model and 99.98% for standard contour (*P* < 0.001), and the original dose distribution showed poor results in automatic CTV segmentation (Fig. 5). These data indicated the final CTV segmentation generated by DL based model remains necessary to be reviewed by senior radiation oncologists rather than geometric values. For the test of agreement, the DL based segmented method has been proven to obtain dose consistency for kidney, bladder and femoral head compared with expert contouring. For dosimetric metrics of OARs, no significant differences were found except for spinal cord and pelvic bone (*P* < 0.001). Point dose such as D_max_ in spinal cord was sensitive to the range of the segmentation in radiotherapy which means the performance of identifying boundaries in DL based model should be improved.

The heatmap of Spearman’s correlation analysis showed that there was no clear strong relationship between geometric metrics and dosimetric differences for most structures (Fig. 5). The only strong correlation was shown for the mean dose of pelvic bone and its 95%HD (R = 0.843, *P* < 0.001). This phenomenon cloud be explained that the dosimetric differences were generated by random noise because of the similar delineation between two methods such as kidney and bladder. Otherwise, the weak link was caused by the segmented reproducibility of DL based model such as CTV and femoral head. However, significant correlation between geometric metrics and dosimetric differences could still be observed due to the inaccurate delineation such as pelvic bone.

In this work, we investigated the performance of DLbased auto segmentation in cervical cancer for patients treated with EBRT. Indeed, as an assisted and efficient tool, automatic approach would relieve physicians from the labor-intensive tasks as well as increase the accuracy and reproducibility of structure delineation.Instead of incorporating a prior knowledge into the process of segmentation that describe as atlas-based segmentation (ABS)^34^, DL based auto segmentation explores the informative representations in a self-learning algorithm and utilizes hierarchical layers of extracted abstraction to accomplish high-level tasks efficiently. Furthermore, in spite of the superior performance of DL based methods on algorithm, the studies are confined mostly to the field of segmentation rather than to establish the workflow solution which have been mentioned above.In other words, DL based methods could play an important role in the complete process of radiotherapy such as “dose prediction”, “toxic prediction” , “efficacy prediction”, etc., segmentation/ “delineation prediction” is only a part of this workflow. Certainly, this work was focus on the question of segmented accuracy which would be a basic part implemented in the workflow of cervical cancer radiotherapy.

Several limitations still exist in our study. First,this work was lack of subjective assessment such as radiation oncologist evaluation or Turing imitation test^35^. Second,the diversity of CT scanner machines,image acquisition protocols, standard contouring,and even tumor staging hampered meaningful comparison of our results with other CNN models. Overall, increasing the amount of training data from different centers using different techniques could make the DL based model more robust, improving the segmentation accuracy.

## Conclusion

This study has demonstrated through both geometric and dosimetric metrics that our DL based auto-segmentation can achieve clinically acceptable contours for most of the EBRT planning structures in cervical cancer patients, although the dosimetric consistency of CTV was a concern. Automatic delineation will be an essential component in cervical cancer workflow which would generate the accurate contouring.

## Data Availability

The datasets used and/or analyzed during the current study available from the corresponding author on reasonable request.
